# Evolutionary applications of electrical stimulation in bone repair: bibliometric trends and temporal dynamics

**DOI:** 10.1530/EOR-2025-0151

**Published:** 2026-07-01

**Authors:** Xiuqing Wang, Jiachen Liu, Yuping Huang, Xiaojing Huang

**Affiliations:** ^1^ Clinical Research Center for Oral Tissue Deficiency Diseases of Fujian Province & Fujian Key Laboratory of Oral Diseases & Fujian Provincial Engineering Research Center of Oral Biomaterial, School and Hospital of Stomatology, Fujian Medical University, Fuzhou, China; ^2^ Stomatological Key Laboratory of Fujian College and University & Institute of Stomatology & Research Center of Dental and Craniofacial Implants, School and Hospital of Stomatology, Fujian Medical University, Fuzhou, China

**Keywords:** electrical stimulation, bone regeneration, bibliometric analysis, expert modeler

## Abstract

- Bone defects cause life-threatening risks and reduced quality of life. Researchers are advancing electrical stimulation technologies through sustained innovation to address these challenges.
- This review used a dual analytical approach integrating bibliometric analysis and expert modeling to systematically evaluate the evolution and trends of electrical stimulation in bone regeneration.
- A total of 2,069 publications from the Web of Science Core Collection met the inclusion criteria.
- Early-stage research focused on electrical properties of bone and electrical stimulation stimulator design. Recent trends show growing emphasis on novel material discovery and optimization for electrical stimulation applications.
- The field was undergoing rapid expansion, engaging growing numbers of countries, institutions, and researchers worldwide.

## Introduction

Endogenous electric field, naturally present in living bone, plays a crucial regulatory role in both skeletal development and regeneration (1, 2). This bioelectrical phenomenon primarily originates from two mechanisms: piezoelectric effect generated by mechanical stress during bone deformation and streaming potential arising from ion flow (3, 4). The piezoelectric property is intrinsically determined by the unique structural organization of biological macromolecules comprising the mineralized collagen matrix of bone (5, 6). Collagen is known for the piezoelectric, pyroelectric, and ferroelectric properties (7, 8, 9). In bone tissue, the combined action of collagen’s non-centrosymmetric molecular structure and hydroxyapatite generates spontaneous polarization and electric potential when bone is subjected to mechanical stress. The absence of stress-induced electric potential in bone disrupts osteocyte-mediated electromechanical signaling, consequently accelerating osteoclastic resorption. The phenomenon is evidenced in both astronauts in microgravity environments and patients during prolonged bed rest (10, 11). Concurrently, bone exhibits a streaming potential phenomenon resulting from ion flow (3), which is characterized by lower amplitude but greater duration compared to the transient piezoelectric effect (12). Those potentials collectively constitute the endogenous electric field of bone and function as signal for regulating the migration and differentiation of bone tissue cells and promoting osteogenesis.

Under pathological conditions, damaged bone tissue exhibits spontaneous generation of endogenous bioelectrical currents, which serve as a critical endogenous stimulus for tissue regeneration (13). When a living bone is injured, two independent currents are generated (14). The initial current is larger, which is related to the piezoelectric property of the bone. Then, a secondary sustained current of lower magnitude emerges, generated by a cellular battery across the injury site. As a result, the injured area exhibits a localized electric field and gradually returns to normal as the fracture heals (15). The bioelectrical field functions as a signal that can adjust the biological behavior of cells in tissue defects, influencing extracellular matrix deposition, cytokine gradients, and cell-to-cell communication (16, 17).

Studies on bioelectric properties of healthy and injured bone tissue have established the therapeutic potential of electrical stimulation (ES) for bone repair. The rising incidence of bone defects severely compromises patients’ quality of life, poses life-threatening risks, and burdens healthcare systems, making ES treatment of great clinical significance (18). This clinical imperative has driven extensive research into its mechanisms and continuous refinement of its applications (19). Current clinical ES is administered by stimulators that deliver capacitive coupling, inductive coupling, and direct current (20). The delivery of ES within an optimal parameter window enhances bone formation and angiogenesis by modulating cellular differentiation and gene expression, thereby aiding repair and reducing healthcare burdens without causing osteonecrosis (21). While ES is safe and effective in clinical applications, some drawbacks of stimulators, such as cumbersome wearability, skin irritation, and heavy device weight, still cause inconvenience for patients. To address these limitations, ES equipment is evolving toward intelligent designs and lightweight solutions (22). And many bioactive materials have been discovered to promote bone regeneration, such as conductive, piezoelectric, pyroelectric, and triboelectric biomaterials (23).

**Figure 1:**
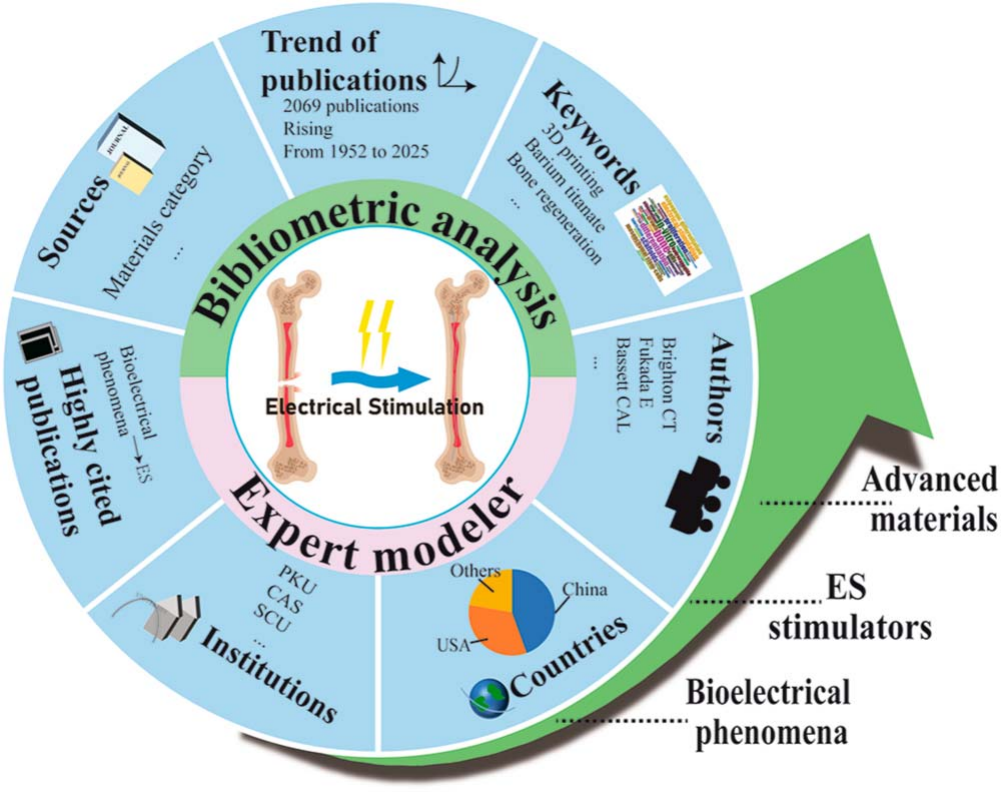
A scheme summarizing different aspects in the dual analysis.

Against the above backdrop, this review introduced the evolutionary application methods of ES and some emerging trends by utilizing bibliometric analysis and time series modeling and forecasting (Fig. 1). Nowadays, bibliometric analysis is widely used to summarize the existing research results, predict the future research direction, and evaluate the contributions of authors, journals, institutes, and countries by using quantitative statistics (24). A time series is data listed in time order. A variety of time series models have been developed to understand the information behind these data, such as moving average, weighted moving average, and single exponential smoothing (25). Through the publications retrieved from Web of Science Core Collection (WoSCC), this review explored technological transformations in ES for bone defect treatment inspired by endogenous bone bioelectricity. It aimed to identify current research focuses and key contributors and assist scholars in recognizing clinical priorities and future research directions by tracking evolution in ES techniques. Moreover, we also hoped to offer a novel and repeatable research path that can be applied to other scenarios.

## Materials and methods

### Data collection and analysis

**Figure 2:**
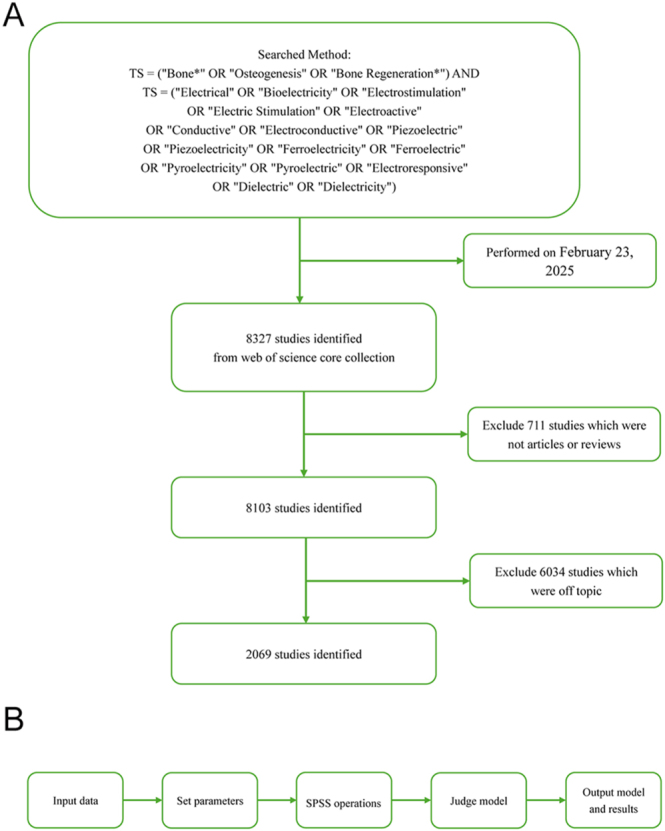
Overall flowchart of article inclusion and expert modeler. (A) Flowchart of the screening process for related publications. (B) Flowchart of the time series modeling and forecasting.

Web of Science (WoS) is one of the most widely accessed academic databases and currently has indexed more than 12,000 journals, 148,000 conference proceedings, and 30,000 books published (26). WoSCC has stricter rules for the inclusion of journals, so the literature retrieved is of high academic quality and credibility. Therefore, bibliometric analysis studies frequently select WoSCC as their primary database (27, 28). In this cross-sectional study, the type of publication was restricted to ‘articles’ and ‘reviews’ from WoSCC, and collected on February 23, 2025. We selected relevant publications and saved them in plain text format for further analysis. These files included complete records and cited references. The search strategy was set as follows: (TS = (‘Bone*’ OR ‘Osteogenesis’ OR ‘Bone Regeneration*’) AND TS = (‘Electrical’ OR ‘Bioelectricity’ OR ‘Electrostimulation’ OR ‘Electric Stimulation’ OR ‘Electroactive’ OR ‘Conductive’ OR ‘Electroconductive’ OR ‘Piezoelectric’ OR ‘Piezoelectricity’ OR ‘Ferroelectricity’ OR ‘Ferroelectric’ OR ‘Pyroelectricity’ OR ‘Pyroelectric’ OR ‘Electroresponsive’ OR ‘Dielectric’ OR ‘Dielectricity’)). The basic process of data collection and retrieval strategy is shown in Fig. 2A.

Inclusion criteria:

- Publications whose titles, abstracts, or keywords contained the above terms.
- Document types were restricted to articles and reviews.
- The records provided complete publication details, including author information, affiliations, abstracts, and cited references.
- The records were directly relevant to the application of ES technology (e.g. using piezoelectric materials) for bone regeneration.

Exclusion criteria:

- Publications that were letters, comments, editorials, or errata.
- Records with incomplete information or those for which the full text was unavailable.
- Studies in which ES was not actually applied for regenerative purposes (e.g. studies on electrosurgical knives for bone cutting).

### Bibliometric analysis

Following data collection, bibliometric analysis was used to monitor the development and patterns of publications, analyze keywords, evaluate current hot topics, and predict future trends (29, 30). The paper also separately analyzed the authors, countries, institutions, journals, and high-impact literature, providing a visual analysis of the main information of ES related to bone and osteogenesis.

The literature collection was imported to CiteSpace version 6.4.R1, VOSviewer version 1.6.20, and Bibliometrix R package version 4.3.2 for analysis. We used CiteSpace version 6.4.R1 to perform a keyword citation burst analysis, which allowed us to identify the frontier areas and development trends of the field. VOSviewer version 1.6.20 was used to create a variety of maps, such as keyword co-occurrence maps, institutional co-authorship analysis, and author co-citation maps. These maps were then used to identify research hotspots and relationships. We also used Bibliometrix R package version 4.3.2 to get basic information about the publication, create a map of the three-field plot of the countries, keywords and affiliations, and analyze countries’ collaboration and production.

### Time series modeling and forecasting

The expert modeler is an efficient and convenient modeling tool provided by SPSS version 27. It was used to provide reliable evidence to predict the future of this field by assessing the volume of publications (31, 32). The model was based on the number of articles in previous years and the best model was automatically selected according to the internal statistical algorithm. The expert modeler employs an iterative algorithm that applies appropriate transformations, such as differencing to achieve stationarity, specifies candidate model structures (e.g. ARIMA), and estimates parameters using optimization methods including maximum likelihood, least squares, and gradient descent. Model selection is subsequently performed using information criteria and goodness-of-fit statistics to identify the optimal forecasting equation. The flow of statistical analysis and prediction is shown in Fig. 2B. The constructed model was subsequently validated using two approaches: *R^2^* and stationary *R^2^* were used to assess the goodness of fit, and the Ljung-Box test was employed to examine residual autocorrelation.

## Results

### Data statistics and aggregate analysis

**Figure 3:**
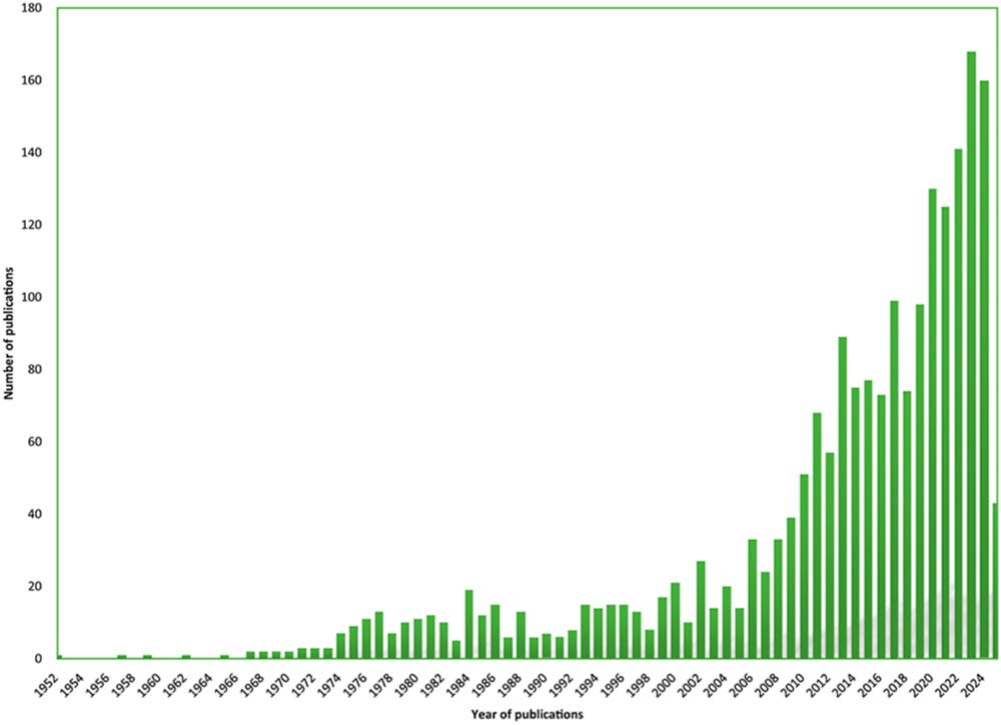
Number of articles published each year.

A total of 8,327 publications were initially retrieved. After discarding publications that failed to meet formatting standards and thematic relevance criteria, 2,069 publications were eligible, covering the period from 1952 to 2025 (Fig. 3). The annual number of publications had increased significantly since the beginning of the 21st century, particularly exceeding 100 articles per year over the past five years (considering that the research deadline was February, the trend of publications in 2025 remained unclear until the end of the year).

### Analysis of keywords

#### The co-occurrence analysis of keywords

**Figure 4:**
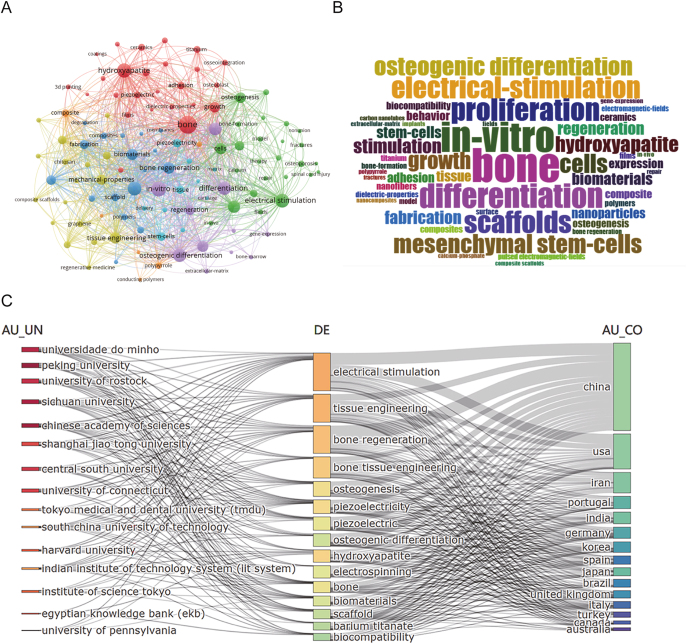
Analysis and visualization of keywords. (A) The co-occurrence analysis of keywords. (B) The wordcloud of keywords. (C) The three-field plot of institutions, keywords, and countries.

Keywords directly reflect research hotspots and main topics. The co-occurrence network built with VOSviewer provides mapped identification of research hotspots. The node size reflects the frequency of keyword, and the line thickness indicates the strength of co-occurrence between two keywords. There were 6,713 keywords, and the minimum frequency of keyword occurrence was set to 25. Meeting the threshold, a total of 111 words are shown in Fig. 4A. All keywords were grouped into seven clusters based on their similarities. Bone (336 occurrences, 1,377 total link strength) appeared the most frequently, followed by *in-vitro* (243 occurrences, 1,292 total link strength), hydroxyapatite (222 occurrences, 986 total link strength), electrical stimulation (219 occurrences, 845 total link strength), and scaffold (211 occurrences, 1,212 total link strength). Furthermore, the wordcloud revealed the prominent themes within the research domain (Fig. 4B).

#### The citation burst analysis of keywords

**Figure 5:**
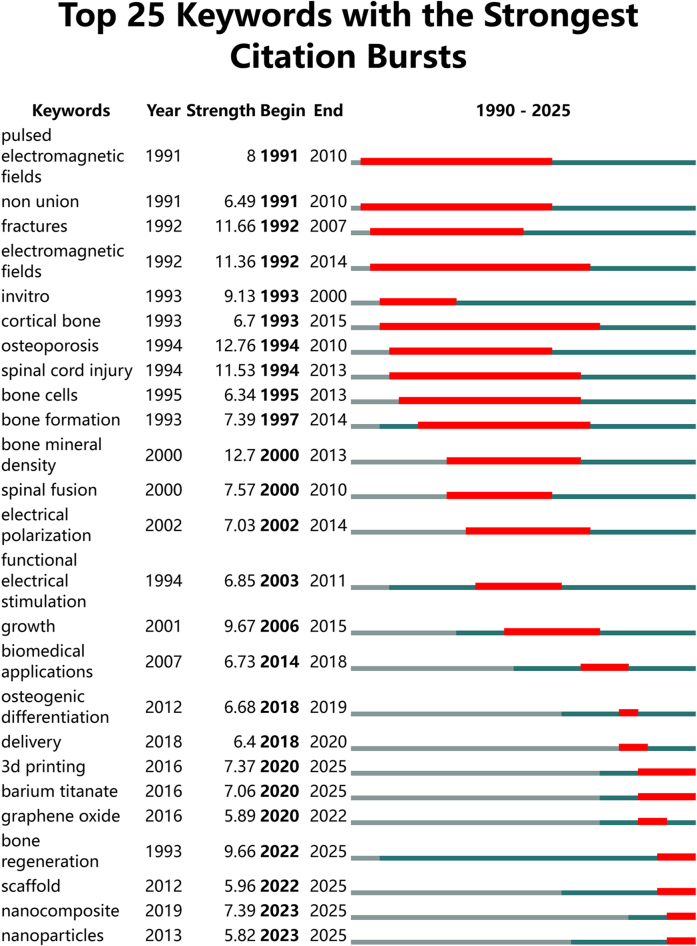
Top 25 keywords with the strongest citation bursts.

CiteSpace was used for keyword citation burst analysis to understand the research hotspot and research trend in a certain period of time. Figure 5 presents the top 25 keywords with the strongest citation bursts and shows the temporal dynamism in the field. The dark blue line represents the citation duration, and the red line shows the burst range of the citation duration. The pulsed electromagnetic fields (strength = 8) received the earliest attention. In the exploration of recent research trends, 3D printing (strength = 7.37), barium titanate (strength = 7.06), bone regeneration (strength = 9.66), scaffold (strength = 5.96), nanocomposite (strength = 7.39), and nanoparticles (strength = 5.82) were keywords that continued until 2025. This demonstrated that research strategies in electrically stimulated osteogenesis shifted from conventional ES applications to advanced bioactive material-based ES approaches.

#### Three-field plot of the countries, keywords, and affiliations

As shown in Fig. 4C, the left field represents institutions, the middle field shows keywords, and the right field shows countries. The number of items was set to 15. The three-field plot visually displayed data flow, energy conversion, and resource allocation through flow paths and flow widths, transforming abstract data into visual widths and exposing the dominant path in the field. The words that were mentioned mainly were electrical stimulation, tissue engineering, bone regeneration, and piezoelectricity. China and the USA contributed to almost all aspects, followed by Iran, Portugal, India, and so on.

### Analysis of authors

**Figure 6:**
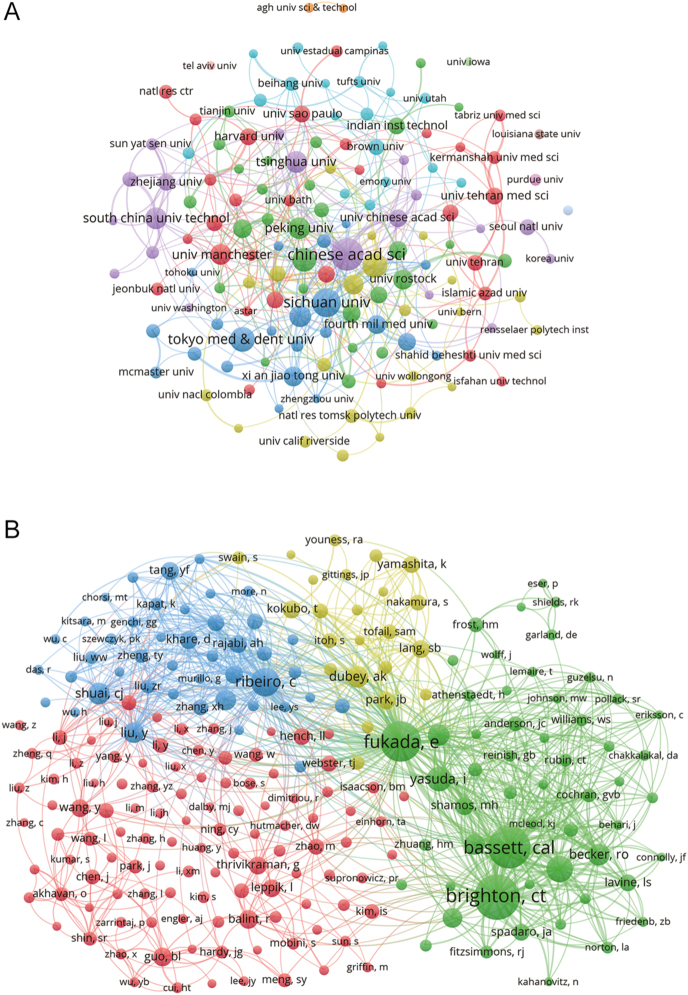
Analysis and visualization of authors and institutions. (A) The co-citation network of authors. (B) The co-authorship network of institutions.

Authoritative authors and knowledge associations in the field can be identified using VOSviewer for co-citation analysis. The minimum number of citations was set to 40, and 231 authors met the threshold. The node size corresponds to the author’s scholarly impact, and the color coding reflects the distribution of researchers across different academic domains. According to Fig. 6A, the co-citation network was divided into four clusters. The three authors in the green cluster, Brighton CT, Fukada E, and Bassett CAL, showed the highest link strength and citations.

### Analysis of countries/regions

**Figure 7:**
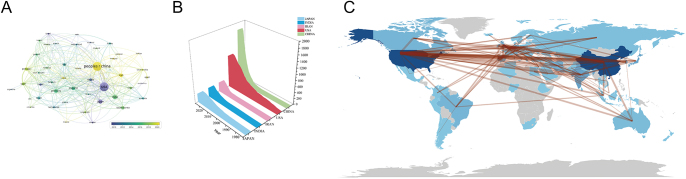
Analysis and visualization of countries. (A) The co-authorship network of countries. (B) The countries’ production over time. (C) Countries’ Collaboration World Map.

The country of the corresponding author was analyzed to reflect the leading country and the country’s dominant position in some fields. SCP (single-country publications) and MCP (multiple-country publications) were separately analyzed. As shown in Table 1, China and the USA were the top two countries, ranking first and second. The countries’ production means the total number of articles of the country, reflecting the breadth of international cooperation. Figure 7B shows the temporal trend in publication output from the top five contributing countries. The USA played a pioneering role in this field, laying its early foundation. This leadership lasted until 2022, when China’s output shifted the landscape. In 2025, China ranked first with 1,777 documents, 67% higher than the USA (1,067 documents).

**Table 1:** The country of the corresponding author.

Country	Articles	Articles %	SCP	MCP	MCP %
China	484	23.4	400	84	17.4
USA	382	18.5	330	52	13.6
India	116	5.6	89	27	23.3
Japan	107	5.2	97	10	9.3
Germany	82	4	64	18	22
Iran	78	3.8	59	19	24.4
South Korea	67	3.2	51	16	23.9
United Kingdom	63	3	35	28	44.4
Brazil	57	2.8	41	16	28.1
Portugal	52	2.5	20	32	61.5

The time-overlapping analysis of country co-authorship in Fig. 7A reveals a collaborative network where China and the USA are central. This finding is further supported by Fig. 7C, which demonstrates their sustained centrality. The colors range from dark blue to light green to bright yellow, representing the average active years of these countries. Interestingly, in the early years, the USA was the leading country, and in recent years, it has changed to China. The countries with a large number of publications also had high rates of cooperation, highlighting the necessity for international collaboration and knowledge sharing in the research process.

### Analysis of institutions

Table 2 lists the ten most productive institutions. The Peking University (108 publications) published the most articles in this field, followed by the Chinese Academy of Sciences (96 publications) and Sichuan University (90 publications).

**Table 2:** The ten most productive institutions.

Ranking	Affiliation	Articles	Country
1	Peking University	108	China
2	Chinese Academy of Sciences	96	China
3	Sichuan University	90	China
4	Universidade Do Minho	84	Portugal
5	University of Rostock	82	Germany
6	Central South University	65	China
7	University of Connecticut	63	USA
8	Shanghai Jiao Tong University	58	China
9	Egyptian Knowledge Bank (EKB)	56	Egypt
10	Harvard University	56	USA

The co-authorship analysis of institutions provides insights into the core institutions and their partnerships. We used VOSviewer to build institutions’ co-authorship network. The minimum number of documents was set to 6, and 147 institutions met the threshold. All institutions were divided into 12 clusters represented by different colors (Fig. 6B). The purple cluster centered on the Chinese Academy of Sciences (91 total link strength), the yellow cluster centered on Universidade do Minho (45 total link strength), and the blue cluster centered on Sichuan University (31 total link strength).

### Analysis of highly cited publications

The citations of publications in a particular field can reflect the impact of the research. Table 3 lists the 10 most cited articles on ES for osteogenesis. The most cited article (33) reached 2,245 citations. The two most cited articles both described the electrical properties of the human body, with the most cited describing dielectric property (33) and the other describing piezoelectric property of bone (1).

**Table 3:** The ten most cited articles.

Ranking	First author (ref)	Year	Journal	DOI	Total citations	TC per year
1	Gabriel C (33)	1996	*Phys Med Biol*	10.1088/0031- 9155/41/11/001	2,245	74.83
2	Fukada E (1)	1957	*J Phys Soc Jpn*	10.1143/JPSJ.12.1158	1,138	16.49
3	Zhang LJ (38)	2009	*Nano Today*	10.1016/j.nantod.2008.10.014	840	49.41
4	Henkel J (39)	2013	*Bone Res*	10.4248/BR201303002	654	50.31
5	Bassett CAL (40)	1962	*Science*	10.1126/science.137.3535.1063	612	9.56
6	Guo BL (58)	2018	*Biomacromolecules*	10.1021/acs.biomac.8b00276	561	70.13
7	Chorsi MT (59)	2019	*Adv Mater*	10.1002/adma.201802084	554	79.14
8	Shin SR (60)	2016	*Adv Drug Deliver Rev*	10.1016/j.addr.2016.03.007	511	51.10
9	Zhao X (61)	2015	*Acta Biomater*	10.1016/j.actbio.2015.08.006	475	43.18
10	Rajabi AH (62)	2015	*Acta Biomater*	10.1016/j.actbio.2015.07.010	435	39.55

### Analysis of article output and impact of sources

Table 4 shows the top ten sources with the highest number of publications in the field. *ACS Applied Materials & Interfaces* (42 articles) was number one, followed by *Journal of Biomedical Materials Research Part A* (41 articles), *Ceramics International* (33 articles), *Biomaterials* (29 articles), and *Journal of Biomechanics* (29 articles). According to the analysis, materials research was the predominant field, producing the greatest volume of publications.

**Table 4:** The top ten sources.

Ranking	Sources	Articles
1	ACS Applied Materials & Interfaces	42
2	Journal of Biomedical Materials Research Part A	41
3	Ceramics International	33
4	Biomaterials	29
5	Journal of Biomechanics	29
6	Materials Science & Engineering C-Materials for Biological Applications	27
7	Clinical Orthopaedics and Related Research	25
8	Journal of Materials Chemistry B	25
9	Journal of Orthopaedic Research	25
10	Journal of Biomedical Materials Research Part B Applied Biomaterials	24

### Expert modeler

We used the entire time span to create a predictive model and later found that pre-20th-century data made the model fit the data poorly. In order to predict the research situation to 2040 and take into account the change of the research situation after the 20th century, the data after 2000 (including 2000) were selected to build the prediction model.

The goodness of fit of the time series model constructed by expert modeler was evaluated using the *R^2^*, stationary *R^2^*, and Ljung-Box test. These results are shown in Table 5. It was appropriate to arrive at values *R^2^* = 0.739 and stationary *R^2^* = 0.909, which meant that the model predicted the data with a high degree of accuracy. The Ljung-Box test was used to test the randomness of the residual errors of the time series model. When the statistical significance (Sig.) value of the test is >0.05, the data are randomly distributed, indicating that the fitting quality of the original model is good. The Sig. was 0.526 > 0.05, which indicated that the residual distribution of the model was random, or the residual sequence was classified as a white noise sequence.

**Table 5:** Test results of the predictive ability of model.

Parameter	Values
Model	Articles
Number of predictors	0
Model fit statistics	
Stationary *R^2^*	0.739
*R^2^*	0.909
MAE	11.976
Ljung-Box Q (18)	
Statistics	15.963
DF	17
Significance	0.526
Outliers, *n*	0

**Figure 8:**
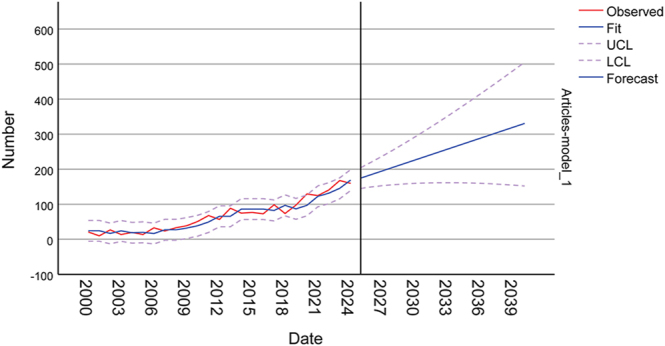
Forecast of future publication volume.

Upper confidence limit (UCL) means the highest range that can be predicted with 95% confidence interval, and lower confidence limit (LCL) means the lowest range. As shown in Table 6 and Fig. 8, the volume of research on the effects of ES on bone is projected to maintain significant growth in the coming years.

**Table 6:** Forecast of future publication volume.

Year	Forecast	UCL	LCL
2025	175	204	145
2026	185	220	149
2027	195	237	153
2028	205	255	156
2029	216	274	158
2030	226	293	160
2031	236	312	161
2032	247	332	162
2033	257	352	162
2034	267	373	162
2035	278	394	161
2036	288	415	160
2037	298	437	159
2038	309	460	157
2039	319	482	156
2040	329	505	153

## Discussion

### Bibliometric information analysis

A total of 2,069 publications on the application of ES in bone and osteogenesis were published from 1952 to 2025, and a significant upward trend was observed over time. The results of the analysis revealed a growing interest in the application of ES in bone and osteogenesis. More specifically, the sharp increase in the number of published papers over the past five years reflected a rapidly growing research interest. The expert modeler’s projection, which extrapolates from the historical growth pattern, suggests that if the current trajectory continues without major disruption, the annual publication volume could reach approximately 329 papers by 2040. This potential growth is hypothetically linked to factors such as the rising global incidence of bone defects and sustained research investment, though these are not variables within the model itself (34, 35). The keyword co-occurrence network delineated a research ecosystem firmly rooted in tissue engineering and biomaterial development, as evidenced by the high frequency and interconnectivity of terms such as scaffold, hydroxyapatite, and *in*-*vitro*. This configuration reflected the field’s historical and ongoing commitment to developing osteoconductive platforms that mimic the extracellular matrix and support cellular proliferation. Keyword citation burst analysis signaled a shift from static, off-the-shelf scaffold designs toward rationally engineered, functionally optimized biomaterials capable of integrating more seamlessly with host tissue.

The co-citation analysis identified Brighton CT, Fukada E, and Bassett CAL as the most influential authors in the field, as evidenced by their highest link strength and citation counts. These pioneering researchers laid the foundational understanding of how ES promotes bone regeneration. For researchers entering or advancing in this domain, closely following the work emerging from these authors’ laboratories and their collaborative networks offers a direct pathway to engaging with the most influential research currents and identifying high-impact directions for future investigation. Research output is influenced by a multifaceted interplay of factors, such as governmental funding allocations, scholarly capacity, and institutional support systems (36). Consequently, under the influence of multiple factors, China and the USA were the most impactful nations. Correspondingly, among the top ten most productive institutions, six were from China and two were from the USA. The two nations fostered extensive collaboration, underscoring the critical role of international partnerships in driving scientific progress.

Citation analysis serves as a powerful bibliometric tool to evaluate a publication’s disciplinary significance by quantifying its frequency of being cited. Through citation analysis, researchers can discern the core literature. Meanwhile, analyzing the publication of highly cited articles enables researchers to trace historical trajectories and identify emerging trends within a field (37). The most cited article (2,245 citations) was a review article that comprehensively evaluated research progress on tissue dielectric properties through 1996 (33). With 1,138 citations, the second most cited article (by Fukada & Yasuda) provided a detailed characterization of bone’s piezoelectric behavior (1). These results provided validation that bone’s electrical properties constitute the biophysical basis for successful ES treatments. Ranking third in citations was a review article (840 citations) that systematically documented the advantages of nanomaterials for bone regeneration. The review demonstrated the capacity of nanomaterials to effectively stimulate osteogenesis and discussed their application in tissue engineering (38). The fourth most cited article (654 citations) was published in 2013 (39). This review provided a systematic evaluation of the current state of biomaterials while offering forward-looking perspectives on next-generation bone regeneration technologies. This demonstrated that advancements in materials science became pivotal to bone regeneration research. The fifth most cited article (612 citations) reported mechanically induced electrical polarization in bone tissue, which established the foundational principles of bone piezoelectricity (40).

By analyzing high-impact journals, researchers can identify the most relevant journals within their field (29). Materials science journals constituted the predominant share of publications, occupying all top five positions by volume. Therefore, researchers may prioritize these journals when investigating this field. The dominance of materials science journals in this ranking further confirmed the central role of advanced materials in the implementation of ES for biomedical applications.

### Implications and new trends

As bone defects caused by an aging population and changing lifestyles become increasingly severe (41), the demand for new technologies and new materials is urgent. The treatment of bone defects is undergoing an evolution from structural reconstruction to intelligent, functionally regenerative systems. This progress has, in turn, attracted a growing number of scholars and clinicians to participate. Two key frontiers drive this evolution: digital-guided design and biomaterial-driven regeneration.

Advanced manufacturing and computational design are enabling precision ES and offer viable solutions for patient comfort, data-driven parameter control, and personalized treatment planning. Through computational modeling of defect geometry, 3D printing enables the fabrication of patient-specific implants that precisely match the anatomical contour (42, 43). Moreover, by computationally optimizing the ratio of bioactive components, such as conductive hydrogels, these 3D printing implants can be further engineered to ensure conformability to tissue defects during movement (44, 45). Beyond structural design, deep learning and machine learning algorithms enable the analysis of omics and imaging data to elucidate the molecular and cellular mechanisms of bone repair, thereby advancing the understanding of the healing process. Another key capability lies in AI algorithms trained on clinical inputs such as injury duration and defect severity, which can assist clinicians in determining patient-specific stimulation parameters, including intensity and treatment duration (46). Overall, as a transformative tool, artificial intelligence assists clinical decision-making by uncovering latent features from injury data, rationally guiding biomaterial design, and forecasting healing trajectories, thereby enabling a shift from empirical practice toward data-driven precision medicine (47, 48).

The second frontier, biomaterial-driven regeneration, is enabled by material innovations. Self-powered piezoelectric materials such as barium titanate, combined with advanced manufacturing techniques, can harvest biomechanical energy from physiological motion, eliminate the need for external batteries and wired connections, and reduce device weight for untethered, long-term applications (23, 49). The integration of self-powered materials with osteogenic components such as hydroxyapatite, calcium phosphate, or bone morphogenetic proteins at optimized ratios acts synergistically to promote bone formation (50, 51). Moreover, hydrogels and conductive nanomaterials serve as ideal interface layers for ES devices, providing soft, tissue–conformable interfaces that reduce mechanical irritation and discomfort during stimulation (52, 53, 54). These advanced biomaterials promote regeneration by imitating the native tissue microenvironment, functioning as osteoconductive scaffolds, or as biocompatible, conformable interfaces that improve the clinical usability of external ES devices (55, 56). Ultimately, realizing the full potential of these frontiers demands a partnership among clinicians, computational experts, and biomaterial researchers to translate technological innovation into patient-centric care.

### Limitations

However, after interpreting the results of the present study, some limitations should be discussed. All publications were primarily retrieved and collected from the WoSCC database. Although WoSCC is the most commonly used and authoritative comprehensive database, several related publications not included in these databases may be missed, resulting in incomplete literature research to some extent. Moreover, while our analysis identified new trends in ES research, several dimensions remain underexplored, including emerging paradigms in 3D bioprinting technology and the translational applications of novel biomaterials in osteogenic contexts. Finally, a limitation of the expert modeler – and indeed any forecasting approach – is that it must simplify reality into a formula, whether intricate or parsimonious. Yet the course of real-world events is shaped by forces more complex and contingent than any equation derived from past observations can fully capture.

## Conclusion and future perspectives

This review systematically evidenced a growing interest in the application of ES in bone and osteogenesis, highlighting the positive impact of ES on bone defect regeneration. The evolution of ES research originated from the discovery of bone’s endogenous bioelectric fields, which inspired therapeutic electrical interventions. This field has progressively advanced toward developing advanced biomaterials. Substantial growth in publication volume reflected escalating recognition of this field’s scientific and translational significance. We anticipated expanding participation in global research from diverse nations, institutions, and interdisciplinary researchers. This broadening engagement will accelerate research activity and drive future innovation.

Despite the demonstrated osteogenic potential of ES in bone regeneration, several critical challenges need to be addressed to pave the way for safe clinical translation. These include elucidating underlying mechanisms, optimizing stimulation parameters, and overcoming translational barriers. First, although ES accelerates osteogenesis through recognized mechanisms such as calcium signaling potentiation, the complete signaling network remains elusive (57). Future studies should employ an integrated methodology combining genomic and proteomic profiling, histomorphometric analysis, and multi-omics to systematically unravel the physiological mechanisms of ES-induced bone formation. Second, achieving patient-specific ES regimens requires the systematic aggregation and analysis of precise, diverse clinical datasets. Integrating experimental evidence with globally sourced clinical outcomes will be crucial for developing tailored stimulation parameters aligned with precision medicine. Third, despite being a transformative force in medicine, artificial intelligence’s role in bone defect treatment remains constrained by limited datasets, insufficient integration of multi-scale healing factors, and poor model interpretability. Addressing these limitations requires global collaboration to establish shared, multicenter databases that expand training data of the model, alongside deeper integration of computational and clinical expertise to develop interpretable, patient-tailored systems. Finally, clinical translation of these findings remains limited. A multidisciplinary framework engaging clinicians, researchers, engineering enterprises, and regulators is essential to systematically assess safety and facilitate clinical translation.

## ICMJE Statement of Interest

The authors declare that they have no known competing financial interests or personal relationships that could have appeared to influence the work reported in this paper.

## Funding Statement

This work was supported by the Joint Funds for the Innovation of Science and Technology, Fujian Province (No. 2021Y9026).

## Author contribution statement

XW and JL were involved in conceptualization, methodology, formal analysis, writing the original draft, and writing – review and editing. YH was involved in writing – review and editing. XH was involved in supervision, funding acquisition, and writing – review and editing.
